# Authors’ Response to Peer Reviews of “Impact of Weekly Community-Based Dance Training Over 8 Months on Depression and Blood Oxygen Level–Dependent Signals in the Subcallosal Cingulate Gyrus for People With Parkinson Disease: Observational Study”

**DOI:** 10.2196/67815

**Published:** 2024-12-13

**Authors:** Karolina A Bearss, Rebecca E Barnstaple, Rachel J Bar, Joseph F X DeSouza

**Affiliations:** 1 Department of Psychology, Algoma University, Brampton, ON, Canada; 2 Dance Studies, York University, Toronto, ON, Canada; 3 Department of Theatre, University of Guelph, Guelph, ON, Canada; 4 Department of Creative Arts, Health and Wellness, University of Guelph, Guelph, ON, Canada; 5 Canada’s National Ballet School, Toronto, ON, Canada; 6 Connected Minds: Neural and Machine Systems for a Healthy, Just Society, York University, Toronto, ON, Canada; 7 Department of Psychology, Centre for Vision Research, Toronto, ON, Canada; 8 Graduate Program in Interdisciplinary Studies, York University, Toronto, ON, Canada; 9 Vision: Science to Applications, York University, Toronto, ON, Canada

**Keywords:** depression, cingulate, GDS, Geriatric Depression Scale, neuroimaging, dancing, Parkinson disease, neurodegenerative, MRI, imaging, neurology, magnetic resonance imaging

*This is the authors’ response to peer-review reports for “Impact of Weekly Community-Based Dance Training Over 8 Months on Depression and Blood Oxygen Level–Dependent Signals in the Subcallosal Cingulate Gyrus for People With Parkinson Disease: Observational Study.”*

## Round 1 Review

We would like to acknowledge and thank our reviewers for taking the time to precisely read and provide constructive feedback and comments on our manuscript titled “Impact of Weekly Community-Based Dance Training Over 8 Months on Depression and Blood Oxygen Level–Dependent Signals in the Subcallosal Cingulate Gyrus for People With Parkinson Disease: Observational Study” [1]. The reviewers’ comments highlighted important areas of concern providing us with the opportunity to address and clarify these within the study. The changes both strengthened and improved the current version of our manuscript and we thank the reviewers for this.

### Anonymous Reviewer [2]

#### General Comments

*I thank the editors for the opportunity to review this article titled “Impact of Weekly Community-Based Dance Training Over 8 Months on Depression and Blood Oxygen Level–Dependent Signals in the Subcallosal Cingulate Gyrus for People With Parkinson Disease: Observational Study.” In this article, the authors report a challenging and well-designed study into the effects of an 8-month dance program designed specifically for reducing nonmotor symptoms in individuals with Parkinson disease on behavioral measures and functional magnetic resonance imaging (fMRI) responses. The article reviews the literature around nonmotor symptoms and the treatment thereof in individuals with Parkinson disease, and the limited existing evidence around the mechanisms of action of these treatments. The authors address the lack of larger-scale studies showing the benefits of dance therapy in this population. The article concludes that dance therapy provides a promising treatment option for nonmotor symptoms in people with Parkinson disease.*

Response: Thank you for your kind words noticing our study was challenging and well designed—our study was indeed very challenging and fun, and we indeed learned a lot about how to run community dance studies longitudinally.

*Below are some comments that the authors may wish to integrate into future revisions of their work.*

#### Specific Comments

##### Major Comments

*1. My main concerns are around the description of the methods used in the study. There is generally not enough detail or justification for decisions made in the acquisition and analysis of the data presented.*

Response: The objective of our preliminary study was to understand how blood oxygen level–dependent (BOLD) signal changes in the subcallosal cingulate gyrus (SCG) relate to changes in depression and mood scores while participating in multisensory interventions such as the Dance for Parkinson Disease (PD) model in people with PD. Modulation of activity in the SCG area has been shown to be associated with changes in depression and mood, where in our study, depression was measured using the Geriatric Depression Scale (GDS). Understanding the relationship between BOLD changes in the SCG area and changes in GDS scores as a function of dance allows us to initially study the neural mechanisms that may provide neuroplasticity in the PD brain related to the reduction in negative mood and increases in positive mood shown in past dance studies using questionnaires. For this study, we now take this one step further by showing a correlation with mood and brain regions in a small but significant group of dancers with PD. We added more to the end of the *Introduction* to the discussion of the SCG and why this node is so important within the emotion circuitry and with respect to our study design.

*2. At the end of the introduction, the “SCG” (subcallosal cingulate gyrus) is mentioned but without further context. As the main finding of the paper rests on the use of the SCG as a region of interest, it would be good to understand more about why only this area was investigated. Were other areas explored/analyzed? If it was the intention to look only at the SCG, why was the field of view so large in the fMRI acquisition? If other areas were looked at, these analyses need to be included. If it was the intention to only look at the SCG, more justification needs to be given as to why this was the only area investigated in the dataset.*

Response: Yes, we focused on this hypothesis of the SCG being an area within the emotion circuit that is used for deep brain stimulation and correlated this with our behavior questionnaires on mood (GDS) that was measured in the dance classes. We have elaborated more on this in the *Introduction* section of the manuscript.

*3. I acknowledge that the sample sizes in the existing literature are of the order n=1, and that a sample of n=10 is a significant improvement on this. However, the description of the sample sizes at each stage of fMRI acquisition is somewhat confused.*

Response: We have provided a flowchart to show the participants scanned and the total number of scans. Hopefully this will further clarify any confusions.

*Did you present the analysis of the healthy control data? What was this used for? Did you present the analysis of the remaining 7 individuals who only completed 1 scan session? How many completed scanning at the baseline session? Perhaps a table or something would be useful to elucidate these numbers less ambiguously.*

Response: The 5 participants that were only scanned with magnetic resonance imaging (MRI) once could not be used for this analysis to compare the change across time in the SCG. Thank you for suggesting to add a table—we added the flowchart showing how many scans were done at each session.

*4. Head motion: could a quantitative comparison be made between the amount of head motion in the people with Parkinson disease group compared to controls? The methods state that no “obvious” motion artifacts were present and that no scans from the people with Parkinson disease group were removed—how was this determined? Was there an objective threshold for what would be excluded? Was motion correction used (and therefore, in what software)? Were any images removed due to motion from the control group?*

Response: There was no head motion that was larger than 2 mm across all 22 scans included in the analysis; this was added to the *Methods* section.

*5. The 30-second “OFF” period seems short and poorly described. What measures were taken to ensure that the participant stopped thinking about the dance or hearing the music playing in their head? Were they instructed to perform another task? How do you know you have not just found that listening to music with positive meaning reduces activity in this region? Was there at least a fixation cross? Please clarify and elaborate on why this is an appropriate design.*

Response: The participants were instructed to imagine dancing to the music they learned to within the community Dance for PD class. We previously used this paradigm with expert ballerinas while they learned choreography over 8 months of ballet performance to see the evolution of modulation with learning and performance in auditory and motor areas (supplementary motor cortex; Bar and DeSouza [3]). We used the 30-second “OFF” period to reset the dancers, and the 18 dancers were all able to stop their imagined dance (DiNota et al [4]). Thus, we were confident that once the music ended, they would not continue imagining the dance since the music ended.

During the no-music period, the whole time, there was was a white fixation cross at the center of the black screen. If their eyes were open, they were instructed to fixate on the cross to not make eye movements to other locations.

*6. There are several points in the Results section where methods are presented (eg, first paragraph of the Results section). Please move all the descriptions of methods into the Methods section and please organize this more logically into behavioral methods (acquisition and analysis) and MRI measures (acquisition, preprocessing, and analysis). No statistical methods are described in the Statistical Analysis section. Please describe here what statistical tests you used (t tests or analysis of covariance?). You state that “no significant interaction was found between experience and GDS”—what test was used? Please outline all statistical tests conducted in the relevant Statistical Analysis section of the Methods section. Which time point was used to determine this? Beginning versus end? Please describe exactly what was done.*

Response: Thank you for this suggestion. We have organized the *Methods* section to separate behavioral methods and MRI methods with the subheadings you have suggested. This could be found on pages 6-7 of the manuscript.

*7. Figure 1: please show which comparisons were significant using asterisks and P values.*

Response: Completed.

*8. Figure 2: I still do not understand the sample sizes used in each of the analyses. Why is B only referring to n=7 people with Parkinson disease—surely you have n=23 for the Geriatric Depression Scale (GDS) data? Then C refers to n=10. This may be resolved by more clearly describing what data were collected in the Methods section as I have requested earlier. But please also display clearly in the format “n=?” on each part of the figure and in the caption how many people were included in that analysis and make clearer throughout why this number is used.*

Response: Thank you for addressing the confusion. We have added a diagram and removed all participants that were not included in the MRI analysis, please refer to our response to question 3 above, which we hope would address the confusion and help answer this question.

We have also removed any participant who were scanned in the MRI but did not fill out the GDS questionnaire at 2 time points and remade Figure 2B, C, and D. Figure 2E did not change since it was the 7 participants that both underwent MRIs and filled out the GDS. We hope this helps with the clarity of this analysis.

##### Minor Comments

*9. In order to fully introduce dance therapy, it would be useful if the authors could refer to and cite some more work assessing the effects of dance therapy on other conditions—I feel that there is a wider bank of evidence for its efficacy in mood disorders and a wider bank of evidence that would give the introduction a more compelling context.*

Response: We have added this specific into the third paragraph indicating that research from [5-7]. The authors used the 16-item Quality of Life Scale from the Oregon Health and Sciences University. This scale is validated for persons with chronic diseases. This scale was used in the study because it aims to measure overall estimate of quality of life, beyond issues only related to health, in addition to incorporating a post–dance class questionnaire of well-being developed by Westheimer [5] and Heiberger et al [6]. Research from Kalyani et al [7] used the Parkinson’s Disease Questionnaire.

*10. Paragraph 3 of the Introduction: “QoL” is mentioned—can the authors briefly add reference to the quality of life measure(s) used in these studies?*

Response: We have added this specific topic into the third paragraph indicating additional research [5-7]. The authors used the 16-item Quality of Life Scale from the Oregon Health and Sciences University. This scale is validated for persons with chronic diseases. This scale was used in the study because it aims to measure overall estimate of quality of life, beyond issues only related to health, in addition to incorporating a post–dance class questionnaire of well-being developed by Westheimer [5] and Heiberger et al [6]. Research from Kalyani et al [7] used the Parkinson’s Disease Questionnaire.

*11. Just above Figure 1, you refer to a previous publication reporting Berg Balance Scale and Timed Up and Go analysis—can you (in the Discussion) compare the effect size reported with the effect size seen in this study?*

Response: We chose to not add this to the manuscript since there are already many manuscripts (which we cite) that have shown that dance helps with motor symptoms and we do not want to add any focus on this in this manuscript. What we hope to show is that using this paradigm of learning and performing dance and visualizing/imagining music in the MRI may in fact help with emotional regulation. We removed that line and associated parts from the revised manuscript.

*12. It is a tiny point, but please refer to an “MR scanner,” not an “MRI scanner.”*

Response: Thank you, this has been changed throughout.

### Anonymous Reviewer [8]

#### General Comments

*The paper is interesting in that it proposes to compare depression scores with task functional magnetic resonance imaging (fMRI) measurements in the subcallosal cingulate gyrus (SCG) of people with Parkinson disease (PD) that underwent dance classes over a long time span (around 8 months).*

*However, it has a major methodological flaw: it correlates depression score changes obtained over 1 day (before and after a dance session) with fMRI signal changes obtained over months. More specifically, it is reported that “A Pearson correlation analysis of the change in GDS data from pre to post (Figure 2B) and the decrease in BOLD signal data showed a strong significant positive correlation…(Figure 2E).” This does not make sense. This correlation should be performed preferably with measures taken at the same time points or, at least, over the same time span.*

Response: Thanks for noticing a potential flaw in our logic! There were errors in our descriptive writing and communication of the methods, but we were *not* using data from only 1 day (before and after a dance session). We are using the change in the GDS across the months for the same participant and their specific BOLD signal change across the months to run the correlations.

We have added better descriptions in the text that describes the GDS reports before and after the last scanning time.

We added Figure 2B’s right panel, which shows the 7 participants’ GDS scores before and after the magnetic resonance (MR) scans.

We have also removed the word “strong” since our pool of participants is only 7 people with PD. This needs to be repeated on a larger population again, which we are hoping to do soon (funding from the National Institutes of Health pending).

#### Specific Comments

##### Major Comments

*1. The correlation between GDS data and blood oxygen level–dependent (BOLD) data should be performed over the same time span.*

Response: Thank you for indicating this flaw in the write-up. We have clarified this issue in the write-up of the *Methods* section. See the description above.

*2. The abstract is misleading since it says that 17 dancers had fMRI scans at 4 time points, but this is not true, since some of those dancers had only 2 or 3 scans. This information (of how many dancers had how many scans) should be in the paper.*

Response: Thank you for your suggestion. The abstract has been updating to include the n values that were used in the study for the analyses. We have revised the mentions of 7 participants used in the *Methods* and *Results* sections, including Figure 2C and D and the figure caption.

*3. I am not sure if it is valid to average the BOLD signals of the participants, as was done in Figure 2C. I would like a better justification for this. Also, it should be reported the number of participants that entered the average of each one of the signals.*

Response: Figure 2C is there to show that the pattern across the 7 participants for the 60 seconds of dance imagery changes across the 8 months, which is only for demonstration purposes. We did not do statistical analysis for Figure 2C’s data. We also removed the very right column of averaged data across the waveforms, since we produced Figure 2D with averages within participants, not across the signals shown in Figure 2C. We use this demonstration because in our previous study (Bar and DeSouza [3]), we show that while expert dancers learn choreography across 8 months, the pattern over prelearning, learning, and performance changes across time.

*4. Figure 2, in general, should be better explained in the text.*

Response: Thank you, we have made additional revisions in the *Results* section to describe this better. Hopefully, it clears up some of the previous unclear points we tried to raise.

*5. Introduction, fifth paragraph: The authors say that “To date, there has been only one fMRI case study with a single participant in which correlations between motor improvements and neural changes were explored.” This is not true—see, for example, [9-12].*

Response: Thank you for bringing these additional articles to our attention and pointing out the mistake of our sentence in detail. We have clarified this sentence to indicate that “To date, there has been only one fMRI case study with a single participant in which correlations between motor improvements and neural changes were explored following dance interventions in people with PD.”

The article by Johansen‐Berg et al [9] used rehabilitation therapy. The Wadden et al [10] study used a sequence-specific motor learning of a perceptuomotor continuous tracking task. Pi et al [11] used high-level basketball players as participants and looked at motor skill learning, especially open skill on the connection patterns. The article by Karni et al [12] used a sequential finger opposition task. None of them used people with PD and neuroimaging tasks.

*6. Still Introduction, fifth paragraph: The authors mention the Batson et al [13] study, but it would be relevant for this study to know with which type of and with how many participants this study was conducted.*

Response: We have added that 1 participant was scanned and compared to the Fullerton Advanced Balance Scale. Thank you for this suggestion.

*7. Still Introduction, fifth paragraph: The authors mention a recent study but they actually do not say if it was conducted with people with PD (this is implicit because they used Dance for PD, but I believe it should be explicitly stated).*

Response: This has now been fixed. Thank you.

*8. Methods: “Study population – Neuroimaging sessions over 8-months”—how come the subsample of 10 people with PD has the same demographic characteristics as the total sample of 23 people with PD?*

Response: This was a mistake, thank you for noticing this error. It has now been appropriately fixed.

*9. Results: a “reduction of GDS scores” is mentioned—I assume that GDS score reduction means improvement in depression symptoms? It would be important to mention this somewhere.*

Response: Thanks, we have added this information to explain what a reduction in GDS scores means. Thank you.

*10. Results: Berg Balance Scale and Timed Up and Go results are mentioned “en passant,” but data are neither shown nor discussed anywhere.*

Response: We have collected other questionnaires as well as the ones mentioned in this study (GDS), but our interest was on mood score changes and any correlation that these changes had to the emotional SCG circuit. The Berg Balance Scale is a measure of balance, and the Timed Up and Go measures performance, lower-extremity function, fall risk, and mobility—these motor results have been successfully published by Bearss et al [14].

##### Minor Comments

*11. Figure 2C: What is the x-axis (variable and units)? Also, the y-axis should be relative (and not percentage) change—or were your maximum changes smaller than 1%?*

Response: The x-axis for Figure 2C is time measured in seconds.

*12. Figure 2D: Same comments as for Figure 2C.*

Response: The x-axis for this is months where we conducted MR scanning.

*13. Figure 2A could be decreased and Figure 2B-E could be increased (I had to set zoom at 400% to be able to see those figures properly).*

Response: Thanks, done!

*14. Introduction, second paragraph: “with efficacy subject to decay over time”—should it be “subjected” instead?*

Response: Indeed, we have changed this accordingly. Thank you.

*15. Methods, Procedures, Imaging: I suggest replacing “slice thick” with “voxels.”*

Response: This has been changed accordingly, thank you.

*16. Methods: In the sentence “Following statistical analysis of the BOLD signal, data was conducted in MATLAB,” I believe it should be “data analysis.”*

Response: Thank you, this has been changed accordingly.

## Abbreviations

BOLD: blood oxygen level–dependent
fMRI: functional magnetic resonance imaging
GDS: Geriatric Depression Scale
MR: magnetic resonance
MRI: magnetic resonance imaging
PD: Parkinson disease
SCG: subcallosal cingulate gyrus
